# Feasibility and Implementation of a Group Therapy Program for Adults Living With Chronic Pain and Sickle Cell Disease

**DOI:** 10.7759/cureus.112599

**Published:** 2026-07-13

**Authors:** Amie Patel, Queen-Ivie C Egiebor, Osatohanmwen Egiebor, Ronnie Blackwell, Felicia Chee, Mary Sehl, Marco Hidalgo, Alice A Kuo

**Affiliations:** 1 Division of Medicine-Pediatrics and Preventive Medicine, University of California Los Angeles David Geffen School of Medicine, Los Angeles, USA; 2 Department of Medicine, University of California Los Angeles David Geffen School of Medicine, Los Angeles, USA; 3 Department of Medicine, University of California, Riverside, Riverside, USA; 4 Department of Psychiatry, Cambridge Health Alliance, Harvard Medical School, Cambridge, USA; 5 Department of Medicine, Division of Hematology/Oncology, University of California Los Angeles David Geffen School of Medicine, Los Angeles, USA

**Keywords:** acceptance and commitment therapy, chronic pain, cognitive behavioral therapy (cbt), group therapy, sickle cell

## Abstract

Adults living with sickle cell disease (SCD) experience high rates of chronic pain, depression, and anxiety, which significantly impair quality of life. Despite this burden, access to mental health services remains limited, and mental health providers often report limited training or apprehension in treating patients with chronic pain. To address these gaps, we implemented a psychotherapeutic skills group co-created and led by clinical psychology postdoctoral residents and tailored for adults with SCD. The program aimed to reduce depression, anxiety, and chronic pain interference in the enrolled patients.

## Introduction

Sickle cell disease (SCD) is the most common inherited blood disorder in the United States, and it results from an autosomal recessive mutation in the beta-globin gene that affects approximately 100,000 people in the United States and millions of people worldwide [1]. Although advances in disease-modifying therapy have improved survival such that most patients now live into adulthood, adults with SCD continue to face substantial chronic disease burden, including recurrent vaso-occlusive pain crises, chronic organ damage, and elevated rates of psychiatric comorbidity [2].

Chronic pain significantly affects over 50% of adults living with SCD. This estimate is drawn from the Pain in Sickle Cell Epidemiology Study (PiSCES), a landmark prospective, multisite cohort study of adults with SCD who completed six months of daily pain diaries; more than half of participants had chronic pain, and one-third had daily or near-daily pain [3,4]. In the same cohort, adult participants reported SCD pain on 54.5% of the days surveyed, and nearly 30% reported pain on more than 95% of days surveyed [3]. Those individuals who suffered from high pain variability with their chronic pain had a worsened quality of life, higher pain catastrophizing, somatic symptom burden, increased health care utilization, and increased opioid use [5].

Depression and anxiety are similarly common among adults with SCD, though reported prevalence varies considerably across studies depending on the assessment method used. In the PiSCES cohort, 27.6% of adult participants met criteria for depression, and 6.5% had an anxiety disorder, and both conditions were associated with greater pain, more healthcare utilization, and increased opioid use [6].

A systematic review of depression in adults with SCD found that reported prevalence ranged from 0% to over 85% across 36 studies, varying substantially based on the psychometric tool used, while other single-site studies have reported depression and/or anxiety in as many as 40% of adult patients [7]. This elevated psychiatric burden, layered onto a disease already marked by recurrent pain, healthcare utilization, and stigma, underscores the need for accessible, disease-specific behavioral health interventions.

Interventions such as cognitive behavioral therapy (CBT) and acceptance and commitment therapy (ACT) have been shown to improve mood and pain catastrophizing [8,9]. A recent multisite randomized controlled trial of digital CBT in adults with SCD-related chronic pain demonstrated improvements in pain interference, depression, anxiety, and quality of life compared to an education control, supporting the disease-specific efficacy of CBT-based approaches in this population [10]. Despite the American Society of Hematology (ASH) recommendation for CBT to reduce the burden of chronic pain in SCD, the use of CBT has been limited due to barriers, including limited availability, stigma, poor engagement, and adherence [11,12].

A 2011 Institute of Medicine report on pain as a public health crisis explicitly called for expanded pain education across health disciplines, including psychology [13]. In direct response, a national multi-stakeholder needs assessment of nearly 2,000 respondents, including psychologists/therapists and psychology training directors, found low self-reported confidence and competence in treating pain, alongside minimal formal pain curricula in graduate training programs [14]. A subsequent review of psychological treatment access for chronic pain similarly identified continuing education and curriculum integration as unmet needs, noting that the American Psychological Association did not begin offering formal psychologist training on chronic pain until 2020 [15]. Therefore, there is a critical need to provide psychology trainees with hands-on experience in chronic pain and chronic illness management in the general population, let alone the sickle cell population.

Taken together, the high prevalence of chronic pain and comorbid psychiatric symptoms in adults with SCD, combined with persistent gaps in provider training and access to evidence-based behavioral interventions, highlights an urgent unmet need for scalable, disease-specific psychotherapeutic programming. A trainee-led, disease-specific group intervention offers one potential model to simultaneously expand patient access to care and build provider competency in this underserved population.

## Materials and methods

Three clinical psychology postdoctoral residents co-created a series of slide decks that eventually guided their implementation of an eight-session group psychotherapy intervention curriculum originally developed by a supervising clinical psychologist. The curriculum integrated CBT, ACT, and motivational interviewing (MI) strategies to build skills in pain acceptance, emotional regulation, activity pacing, and value-based goal setting among adults (>18 years) with SCD. Each session would start with a mindfulness activity. Afterward, each session combined brief didactic instruction on CBT, ACT, and MI concepts with experiential practice, including structured worksheets, guided in-session exercises (e.g., relaxation training, values clarification, and problem-solving walkthroughs), and group discussion; participants also completed weekly homework, such as a pain diary, to reinforce skills between sessions. One psychiatric nurse practitioner with specialization in pain management was available at select group sessions to answer participants’ questions related to medical care. Table 1 shows an overview of the curriculum created for each session. 

**Table 1:** Overview of the eight-session group therapy curriculum for chronic pain management in sickle cell disease ACT, acceptance and commitment therapy; CBT, cognitive behavioral therapy; MI, motivational interviewing.

Session #	Session title	Objectives
1	Introduction to the Group & Foundations of Chronic Pain Management	1) Establish group norms and confidentiality; 2) provide education on chronic pain and its management; 3) introduce CBT, ACT, and MI principles; 4) develop participant goals for the group
2	Cognitive Restructuring and Challenging Negative Thoughts	1) Identify negative thought patterns associated with pain; 2) teach cognitive restructuring techniques; 3) introduce the concept of cognitive defusion from ACT
3	Pain and Emotional Regulation	1) Explore the relationship between emotions and pain; 2) teach relaxation and mindfulness techniques for emotional regulation; 3) encourage mindfulness-based acceptance of emotions and pain
4	Behavioral Activation and Activity Pacing	1) Introduce behavioral activation to break the cycle of avoidance; 2) teach activity pacing to manage energy and prevent flare-ups; 3) link meaningful activity to values
5	Pain Acceptance and Letting Go of Control	1) Teach acceptance strategies for reducing the struggle against chronic pain; 2) introduce cognitive defusion techniques from ACT; 3) link acceptance to meaningful action
6	Values-Based Living and Goal Setting	1) Clarify personal values and how they relate to pain management; 2) help participants set specific, meaningful goals that align with their values; 3) continue linking pain acceptance to value-based actions
7	Coping With Setbacks and Relapses	1) Teach participants how to handle setbacks and pain flare-ups; 2) encourage self-compassion and resilience; 3) develop an individualized relapse prevention plan
8	Consolidation and Maintenance of Pain Management Skills	1) Review key skills learned in the group; 2) help participants develop a long-term pain management plan; 3) celebrate progress and group closure

Every eight sessions were labeled as a round, and a total of three rounds were completed. Each session was conducted weekly over Zoom for 90 minutes. The objectives of the program were to improve psychology trainee competency in helping individuals with chronic pain related to SCD, reduce barriers to access to mental health care, analyze feasibility with participant engagement, and assess any clinical effectiveness of the program. An IRB exemption was obtained to retrospectively review charts of patients with SCD enrolled in the sickle cell program. 

Patients were consented to participate in the program and were encouraged to attend at least four of the eight sessions from any of the three rounds. Since the sessions occurred over Zoom, patients could participate even if admitted inpatient. To increase attendance, standard MyChart automated messages were sent to all participants. During the second round, additional reminder texts were sent and, during the third round, participants who had previously joined but had not logged in to the Zoom session were directly called. Patients completed the Patient-Reported Outcomes Measurement Information System (PROMIS) Pain Interference scoring, Patient Health Questionnaire-9 (PHQ-9), and Generalized Anxiety Disorder-7 (GAD-7) to assess clinical improvement within two months, both before the first session and within two months after completion of the program [16-18]. The PROMIS Pain Interference measure was reproduced for single-use research, as allowed by the terms and conditions of Health Measures (©PROMIS Health Organization) [16]. Psychology trainees were also sent a survey to assess their comfort and competency.

## Results

A total of 21 participants consented to participate in the group therapy program, of whom 15 attended at least one session across three rounds. Seven participants met the engagement goal of attending at least four sessions during any of the three rounds (five attended four sessions, one attended five sessions, and one completed nine sessions). Among the seven participants, 71% were female, and 29% were male; 57% were aged 23-29 years, and 43% were aged 31-37 years. Participant engagement improved notably in the third round, with the highest attendance observed after implementation of direct phone outreach in addition to automated messaging. Average sessions attended per participant nearly doubled, from 1.7 in rounds 1 and 2 to 3.1 in Round 3, and the proportion of participants attending more than one session increased by round 3 from 43% to 33% to 71% (Table 2).

**Table 2:** Participants' engagement during each of the three rounds

Round	Unique participants	Total sessions attended	Avg. sessions/participant	% attendance (of eight possible)	% participants attending >1 session
1	7	12	1.7	21%	43%
2	6	10	1.7	21%	33%
3	7	22	3.1	39%	71%

Among participants with available pre- and post-clinical questionnaire data, the majority demonstrated improvement in mental health outcomes (Table 3). Specifically, four of six (67%) participants showed a reduction in depressive symptoms as measured by the PHQ-9, while one participant remained unchanged and one demonstrated increased scores. Similarly, improvement in anxiety symptoms was observed in four of six (67%) participants with complete GAD-7 data. Two participants demonstrated worsening scores, while no participants had unchanged scores. PROMIS Pain Interference scores were collected but demonstrated variability and were difficult to interpret due to concurrent acute pain episodes and hospitalizations among participants at the time of assessment.

**Table 3:** Summary of changes in PHQ-9, GAD-7, and PROMIS-PI comparing pre- and post-group therapy GAD-7, Generalized Anxiety Disorder-7; PHQ-9, Patient Health Questionnaire-9; PROMIS-PI, Patient-Reported Outcomes Measurement Information System Pain Interference.

Outcome	Improved	No change	Worsened
PHQ-9	4 (67%)	1 (17%)	1 (17%)
GAD-7	4 (67%)	0 (0%)	2 (33%)
PROMIS-PI	2 (50%)	1 (25%)	1 (25%)

Pre- and post-therapy scores were analyzed using Wilcoxon signed-rank tests for paired differences in R with missing data and those with zero change excluded across three outcome measures (Table 4). Descriptive statistics, including mean paired differences and SDs, were calculated for PHQ-9 (n=5), GAD-7 (n=6), and PROMIS Pain Interference (n=3) scores. PHQ-9 scores demonstrated a mean reduction of 4.8 points (SD=3.96) following group therapy (V=14, p=0.104), suggesting a clinical trend toward improvement in depressive symptoms. GAD-7 scores showed a mean reduction of 0.5 point (SD=4.42), which did not approach statistical significance (V=8.5, p=0.844), with high variability across participants limiting interpretation. PROMIS Pain Interference scores improved by a mean of 4.1 points (SD=5.45) among the three participants with complete data, though this also did not reach significance (V=5, p=0.500). No outcome measure reached the threshold of statistical significance (α=0.05), consistent with the pilot nature of this study. A post-hoc power analysis estimated that a sample of 44 participants would be required to achieve 90% power to detect a clinically meaningful difference using a two-sided Wilcoxon signed-rank test. With regards to trainee feedback, one of three trainees completed a brief evaluation of their experience and reported feeling very comfortable helping patients develop coping strategies for chronic pain and using CBT, ACT, and MI skills with patients with complex medical needs, including sickle cell.

**Table 4:** Pre- and post-group therapy mean scores and Wilcoxon signed-rank test results GAD-7, Generalized Anxiety Disorder-7; n, number of participants with complete pre- and post-therapy data included in the analysis for that outcome; PHQ-9, Patient Health Questionnaire-9; PROMIS-PI, Patient-Reported Outcomes Measurement Information System Pain Interference; SD, standard deviation; V, Wilcoxon signed-rank test statistic (sum of positive ranks), calculated in R. Mean change = post-therapy minus pre-therapy score; negative values indicate improvement. p-values are two-tailed at α=0.05. Missing data and those with zero change were excluded per outcome.

Outcome	n	Mean pre (SD)	Mean post (SD)	Mean change (SD)	V	p-value
PHQ-9	5	12.80 (7.01)	8.00 (7.31)	−4.80 (3.96)	14	0.104
GAD-7	6	7.67 (6.92)	7.17 (5.81)	−0.50 (4.42)	8.5	0.844
PROMIS-PI	3	65.80 (2.46)	61.67 (6.79)	−4.13 (5.45)	5	0.500

## Discussion

This pilot retrospective study demonstrates that a virtual psychotherapy group program targeting chronic pain management among adults with SCD in both out- and inpatient settings is both feasible and acceptable, with early signals suggesting improvement in mental health outcomes. These findings align with prior literature demonstrating that behavioral interventions such as CBT and ACT can improve coping, mood, and pain-related distress in individuals with chronic pain, including those with SCD [8,9,19]. Over the course of the program, participants demonstrated increased engagement and a willingness to share personal experiences related to pain and mental health, suggesting that group cohesion and psychological safety developed over time -- an important mechanism underlying the effectiveness of group-based interventions [20].

Although quantitative improvements in PHQ-9 and GAD-7 scores were observed in a subset of participants, interpretation is limited by small sample size and variability in assessment timing. Surveys were completed within a two-month window before and after participation, and several participants were experiencing acute vaso-occlusive episodes or hospitalization at the time of assessment, which may have influenced symptom reporting. Prior studies have similarly highlighted the fluctuating nature of mood and pain symptoms in SCD, which complicates outcome measurement and may obscure treatment effects [3,5]. In addition, increases in symptom scores observed in some participants may reflect improved emotional insight and reduced underreporting rather than true clinical worsening. This interpretation is supported by existing literature describing stigma related to pain in SCD, which can lead to underreporting of psychological distress at baseline [21,22].

Engagement improved across successive rounds, particularly following the implementation of more direct outreach strategies. While automated messaging alone had a limited impact, the addition of text reminders and direct phone calls was associated with increased attendance. This is consistent with prior digital and behavioral health intervention studies in SCD, which demonstrate that multimodal engagement strategies are often necessary to overcome barriers such as competing medical demands, transportation challenges, and psychosocial stressors [11,23]. Furthermore, participants who attended multiple sessions were likely to continue attending, suggesting that early engagement and perceived relevance of content are critical determinants of retention. These findings reinforce the importance of relationship-building and proactive communication in behavioral health program implementation.

From an educational perspective, this model addresses a well-documented gap in psychology training related to chronic pain and complex medical comorbidities. Previous national assessments have identified low confidence and limited formal training among mental health providers in managing chronic pain [14]. In this study, one trainee formally reported increased comfort in applying CBT, ACT, and MI techniques to patients with SCD in chronic pain, possibly suggesting that supervised, disease-specific group interventions may serve as an effective experiential learning model. However, only one trainee completed the post-survey analysis, which limits our conclusion. Expanding such opportunities may be particularly important given ongoing workforce shortages in behavioral health and the increasing recognition of integrated care models for chronic disease management [24].

This study has several limitations. The small sample size, incomplete outcome data, and variable session attendance limit the ability to draw definitive conclusions regarding clinical effectiveness. As a retrospective review without a control group, the study is also subject to selection bias and confounding factors. Additionally, heterogeneity in participation (i.e., varying numbers of sessions attended across rounds) limits assessment of dose-response relationships. Future studies should focus on improving retention strategies, standardizing outcome collection, and incorporating prospective designs with larger sample sizes. Evaluation of additional outcomes, including healthcare utilization, opioid use, and functional status, will be critical to understanding the broader impact of such interventions.

## Conclusions

In this retrospective study of a clinical program, we describe the implementation of a psychology trainee-created and led group therapy program for adults with SCD and chronic pain. This model appears feasible and may improve access to behavioral health services while simultaneously enhancing trainee competency in managing chronic pain and complex medical conditions. Early findings suggest potential improvements in both engagement and mental health outcomes, particularly when active outreach strategies are implemented and participant buy-in is achieved.

Further research is needed to evaluate the effectiveness of this program in larger cohorts and to assess its impact on clinical outcomes, including pain interference, healthcare utilization, and medication use. This scalable model may represent a promising approach to addressing both workforce limitations in behavioral health and unmet psychosocial needs in individuals living with SCD.
